# Colocality to Cofunctionality: Eukaryotic Gene Neighborhoods as a Resource for Function Discovery

**DOI:** 10.1093/molbev/msaa221

**Published:** 2020-09-04

**Authors:** Fatima Foflonker, Crysten E Blaby-Haas

**Affiliations:** Brookhaven National Laboratory, Biology Department, Upton, NY

**Keywords:** genomics, Chlorophyta, phylogenomics, gene function

## Abstract

Diverging from the classic paradigm of random gene order in eukaryotes, gene proximity can be leveraged to systematically identify functionally related gene neighborhoods in eukaryotes, utilizing techniques pioneered in bacteria. Current methods of identifying gene neighborhoods typically rely on sequence similarity to characterized gene products. However, this approach is not robust for nonmodel organisms like algae, which are evolutionarily distant from well-characterized model organisms. Here, we utilize a comparative genomic approach to identify evolutionarily conserved proximal orthologous gene pairs conserved across at least two taxonomic classes of green algae. A total of 317 gene neighborhoods were identified. In some cases, gene proximity appears to have been conserved since before the streptophyte–chlorophyte split, 1,000 Ma. Using functional inferences derived from reconstructed evolutionary relationships, we identified several novel functional clusters. A putative mycosporine-like amino acid, “sunscreen,” neighborhood contains genes similar to either vertebrate or cyanobacterial pathways, suggesting a novel mosaic biosynthetic pathway in green algae. One of two putative arsenic-detoxification neighborhoods includes an organoarsenical transporter (ArsJ), a glyceraldehyde 3-phosphate dehydrogenase-like gene, homologs of which are involved in arsenic detoxification in bacteria, and a novel algal-specific phosphoglycerate kinase-like gene. Mutants of the ArsJ-like transporter and phosphoglycerate kinase-like genes in *Chlamydomonas reinhardtii* were found to be sensitive to arsenate, providing experimental support for the role of these identified neighbors in resistance to arsenate. Potential evolutionary origins of neighborhoods are discussed, and updated annotations for formerly poorly annotated genes are presented, highlighting the potential of this strategy for functional annotation.

## Introduction

The classic, but outdated, view of eukaryotic genomes is of lone genes randomly situated in a sea of noncoding DNA. This picture is derived from the observation that structural organization does not appear to be necessary for coregulating functional units in eukaryotes. In contrast to prokaryotes, where functionally cooperating proteins are often encoded by operons, transcription and translation are uncoupled in eukaryotes. However, as the number of sequenced eukaryotic genomes and transcriptomes has increased, and the function of those encoded proteins has been revealed, nonrandom gene organization has emerged as a characteristic of eukaryotic genomes. In addition to tandem arrays of duplicated genes (Rizzon et al. 2006; Jackson 2007; Despons et al. 2010; Beauchemin et al. 2012), physical clustering of pathway members and coregulated genes have been observed (Lee and Sonnhammer 2003; Michalak 2008). In some cases, the clustering can be explained by the presence of bi-directional genes under the control of a shared promoter (Beck and Warren 1988). However, in other cases, functionally linked gene clusters contain more than two genes each controlled by an independent promoter. Examples include metabolic gene neighborhoods for biotin (Hall and Dietrich 2007), l-rhamnose (Watanabe et al. 2008), nitrate (Quesada et al. 1993), degradation of toxic compounds (Bobrowicz et al. 1997), and natural products (Nützmann et al. 2016).

Systematic identification of gene neighborhoods in eukaryotes typically rely on the availability of gene-function knowledge. These methods combine gene proximity with metabolic pathway reconstruction (Schläpfer et al. 2017), sequence similarity searches of known biosynthetic gene clusters (Vesth et al. 2016; Kautsar et al. 2017; Töpfer et al. 2017), enrichment of common functional roles (i.e., derived from Gene Ontology terms) (Yi et al. 2007; Mihelčić et al. 2019), or coexpression analyses (Foflonker et al. 2016; Vesth et al. 2016; Banf et al. 2019). One early systematic approach to estimating the extent of gene clustering in eukaryotes used metabolic pathways as defined in the Kyoto Encyclopedia for Genes and Genomes. This study found that between 30% and 98% of pathway members in five target species are in closer proximity to one another than would be expected by chance (Lee and Sonnhammer 2003). The popular antiSMASH and plantiSMASH algorithms use gene proximity and HMM-profiles built with known biosynthetic enzyme families to predict biosynthetic gene clusters for secondary metabolites in fungal and plant genomes (Medema et al. 2011; Kautsar et al. 2017). Recently, a more generalized approach for identifying gene clusters, EvolClust, which does not require an a priori understanding of protein family function, identified 12,120 conserved gene clusters from 341 fungal genomes and 8,778 conserved gene clusters from 145 insect genomes (Marcet-Houben and Gabaldón 2020). The widespread occurrence of proximal gene clusters in eukaryotes, many of which have been shown to encode sets of functionally cooperative proteins, points to the prospect of using conserved gene proximity in eukaryotes as a resource for understanding gene function.

Equating conserved colocality with cofunctionality has been a fruitful approach for predicting gene functions in prokaryotes. Overbeek et al. (1999a, 1999b) first formalized this method to predict functional coupling based on conservation of gene clusters across bacterial genomes. Subsequently, this in silico approach to gene function prediction has served as a cornerstone in a suite of comparative genomic tools often referred to as “guilt-by-association,” whereby the function of a gene is informed by the functions of genes it associates with (Aravind 2000; Galperin and Koonin 2000; Shmakov et al. 2019). Examples include finding missing genes for known enzymes (Klaus et al. 2005), genes for alternative pathways (Kurnasov et al. 2003), and finding missing genes for proposed transporters (Rodionov et al. 2009). While such prokaryotic gene-function discovery examples abound, this approach to gene-function prediction has not been widely applied to eukaryotic genomes.

Therefore, we aimed to detect evolutionarily conserved eukaryotic gene neighborhoods and determine whether these clusters could be used to understand gene function. We chose to focus on gene neighborhoods in green algae, photosynthetic eukaryotes in the Viridiplantae lineage with a common ancestor that dates back 972 to 670 Ma (Morris et al. 2018). Green algae are found in both phyla of Viridiplantae: the Chlorophyta (green algae) and Streptophyta (green algae and land plants) (Leliaert et al. 2012). Green algae form a morphologically and phenotypically diverse group with nuclear genomes that can contain anywhere from 7,500 to 18,000 protein-coding genes. Functionally annotating these genomes is challenging because of their evolutionary distance to well-characterized organisms (Blaby-Haas and Merchant 2019). Typically, only 25–50% of predicted proteins in any given algal genome have detectable sequence similarity to defined domains in the Pfam database (Blaby-Haas and Merchant 2019), and only 125 algal genes (out of more than 340,000 sequenced genes) are annotated with an experimentally supported GO term (http://amigo.geneontology.org/; last accessed April 2020).

Here, we surveyed ten green algal genomes to detect evolutionarily conserved gene neighborhoods, independent of coexpression data or functional annotations. A search for Proximal Orthologous Gene (POG)pairs conserved across at least two taxonomic classes resulted in the identification of 317 conserved gene neighborhoods. Assuming that these genes have evolved through vertical inheritance in these algae, gene proximity in some neighborhoods has likely been conserved since the chlorophyte-streptophyte split estimated at 1,000 Ma (Kumar et al. 2017). In addition to capturing previously reported functional gene clusters, we identified uncharacterized neighborhoods of potentially functionally cooperating genes and leveraged phylogenetic relationships, sequence similarity networks, and across-kingdom comparative genomics to generate hypotheses regarding the function of identified gene neighbors. We describe the resulting identification of two novel arsenic-detoxification gene neighborhoods and a putative green algal mycosporine-like amino acid (MAA) biosynthetic cluster. The presence of one of these three gene neighborhoods in *Chlamydomonas reinhardtii*, for which a sequenced mutant library is available (Li et al. 2019), enabled subsequent testing of the predicted role of a novel phosphoglycerate kinase (PGK) in arsenic resistance. Although conserved gene neighborhoods are not common, they can provide an effective source of functional inferences for understanding gene function in algae and beyond. Identification of nonalgal homologs of neighbors suggests de novo assembly of neighborhoods likely formed through genome rearrangement or duplications and neofunctionalization rather than recent horizontal gene transfer (HGT) of intact gene clusters.

## Results

### Identifying Conserved Gene Neighborhoods

We compared gene proximity in the nuclear genomes of ten green algae that have high-quality publicly accessible genome assembles and gene models (see Materials and Methods section). Evolutionarily conserved gene neighborhoods were identified using 1 of 3 criteria: 1) POG pairs conserved in a minimum of 4 of 9 chlorophyte genomes (ensuring POG pairs from at least two taxonomic classes), 2) conserved POG pairs between the chlorophytes and a streptophyte alga, *Klebsormidium nitens* (3 species minimum), potentially indicating conservation over longer evolutionary time, and 3) cooccurring POG pairs conserved in a minimum of three species (i.e., orthologous genes pairs that are proximal in all genomes in which they are present, whereas criteria 1 and 2 allow for orthologous gene pairs to be present, but not clustering with each other, in some genomes). The perl script is available from GitHub (https://github.com/ffoflonker/gene-neighborhoods), and a graphical overview of the method and sample output can be seen in supplementary fig. 1, Supplementary Material online.

**Fig. 1.**
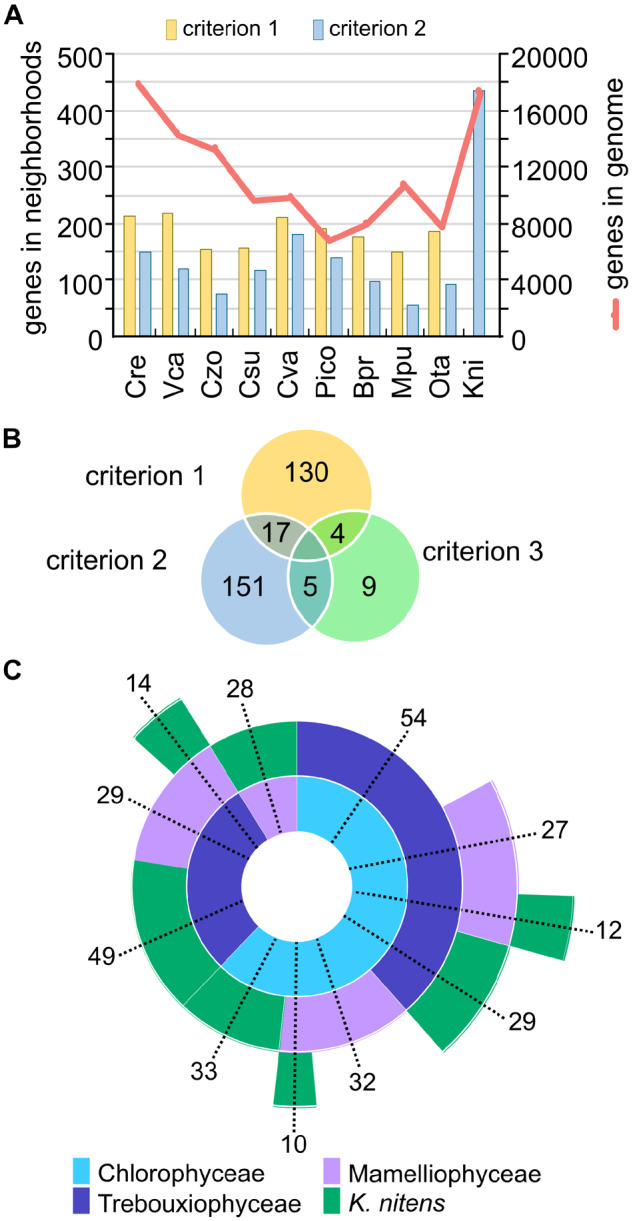
Summary of neighborhood distribution across green algal genomes analyzed. (*A*) Number of gene neighborhoods detected by criterion 1 or 2. (*B*) Number of gene neighborhoods identified in the three criteria. (*C*) Sunburst chart representing the distribution across green algal lineages of gene neighborhoods containing genes from 2 or more taxonomic classes, which met 1 or more criteria. The numbers represent the number of gene neighborhoods with the given taxonomic membership.

Excluding histone clusters, which represent conserved tandem gene arrays, a total of 85, 152, 204, and 189 neighborhoods were found for window sizes 4, 6, 8, and 10, respectively, using criterion 1 (supplementary fig. 2, Supplementary Material online). The number of gene neighborhoods detected began to decrease with increased window size, because the larger gene windows resulted in merging of smaller neighborhoods. We therefore set a conservative window size of 6 genes. The majority of neighborhoods detected with criterion 1 are between 2 and 4 POGs in 4–6 species (supplementary fig. 3, Supplementary Material online).

**Fig. 2.**
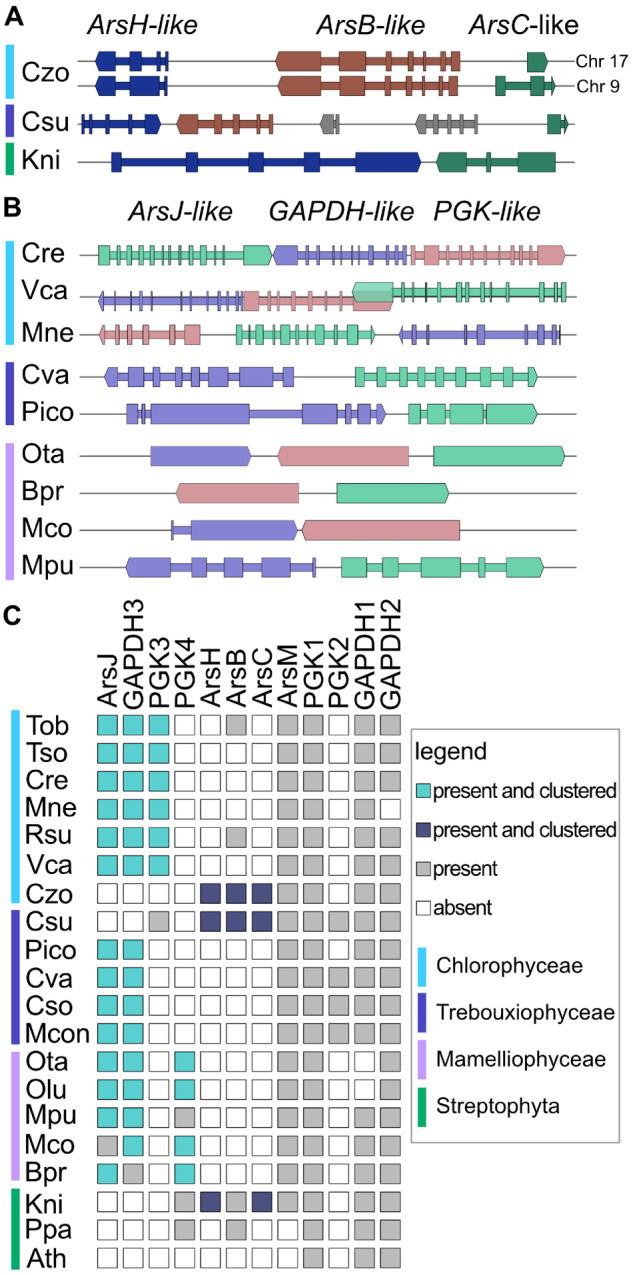
Putative arsenic-detoxification gene neighborhoods. (*A*) Gene clusters of the *ArsH*-type. (*B*) Gene clusters of the *ArsJ*-type. For both panels, thick bars represent exons, thin bars represent introns, and gene models are scaled relative to one another in each genome but not between genomes. Gray gene models correspond to genes not included in the identified neighborhood. (*C*) Phylogenetic profile of cluster members and homologs. For all panels, the colored bars on the left designate taxonomic relationships, as indicated in legend.

**Fig. 3.**
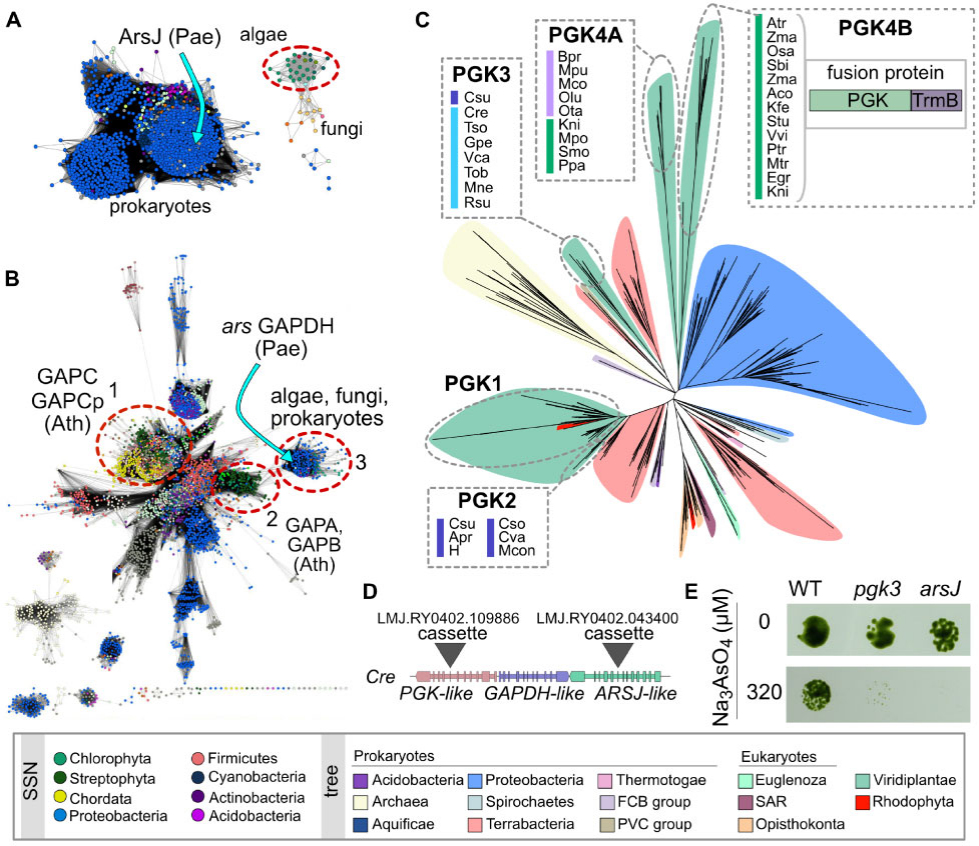
Phylogenomic analysis of ArsJ-, GAPDH-, and PGK-like families. (*A*) Sequence similarity network (SSN) of proteins similar to the MFS-type transporter from algae. The location of the characterized ArsJ transporter node from *P. aeruginosa* (Pae) is indicated. The cluster containing algal homologs is indicated with a dashed red circle. (*B*) SSN of UniRef 90 clusters containing IPR020830. The three clusters containing green algal homologs are indicated with a red dashed outline. The name of enclosed subfamilies from *A. thaliana* (Ath) is given next to the corresponding cluster. The location of the *ars* GAPDH node from *P. aeruginosa* is also indicated. The nodes in panels *A* and *B* are colored by phylum. Predominate phyla colors are given in the legend. (*C*) Phylogenetic reconstruction of the PGK-like family. Background color corresponds to taxonomy according to the legend. Clades containing green algal homologs are circled with a gray dash outline. For clades that are distinct from the canonical PGK1 clade, binomen abbreviations corresponding to leaves are given, and the bar to the left indicates taxonomic group as in figure 2. A cartoon representing the domain fusion found in all indicated land plant homologs is also given for PGK4B-type proteins. (*D*) Location of cassette inserts in CLiP mutants tested for arsenate sensitivity in panel *E*. (*E*) Growth of *C. reinhardtii* wild-type and CLiP mutants under 0 and 320 µM sodium arsenate in TAP media after 5 days of incubation.

Additional neighborhoods were detected with criteria 2 and 3 (fig. 1). A total of 169 neighborhoods were conserved between the streptophyte and chlorophyte algal genomes analyzed (criterion 2). Of these, 17 neighborhoods were also detected with criterion 1 (fig. 1). Using the more stringent cooccurring strategy, 18 neighborhoods were detected containing POG pairs from multiple taxonomic classes. A total of 317 neighborhoods met at least one criterion, with few detected by multiple criteria, indicating the value in utilizing multiple strategies (fig. 1*B*). The gene neighborhoods were shared among the taxonomic classes to varying degrees (fig. 1*C*). We found that regardless of genome size (which ranges from 13 to 131 Mb) or total number of genes (which ranges from 7,367 to 17,741), each genome had roughly the same number of genes conserved in gene neighborhoods (fig. 1*A*). The result is that genomes with smaller gene inventories tended to have a relatively larger number of conserved gene neighborhoods. Due to the definition of search criterion 2 (i.e., all reported neighborhoods must contain the streptophyte alga *K. nitens*), the number of neighborhoods in *K. nitens* may seem high compared to the other green algae (fig. 1*A*).

Our analysis captured conservation of 5 previously reported functional gene clusters involved in urease assembly (Qiu et al. 2013), urea assimilation (Strope et al. 2011), photorespiration (Foflonker et al. 2016), nitrate metabolism (Quesada et al. 1993; Palenik et al. 2007; Foflonker et al. 2015), and Fe-hydrogenase assembly (Cornish et al. 2015) (table 1). For the majority of gene neighborhoods, the potential functional relevance that has driven conservation of membership is unknown. Either functional annotations are unavailable (i.e., genes of unknown function), the available functional annotations are vague (e.g., hydrolase), or a functional link between neighbors is not readily apparent. Therefore, lists of gene neighborhoods were ranked, giving weight to smaller orthologous groups, and manually curated. Notable potentially functionally relevant gene neighborhoods can be seen in table 1; the full outputs are available in supplementary file 1, Supplementary Material online. Potentially functionally relevant gene neighborhoods consist of a variety of different putative functions, including genes involved in nitrogen recycling, chaperones, H_2_ production, oxidative stress responses, and detoxification. Two putative arsenic-detoxification neighborhoods and a putative MAA gene cluster are described in more detail below.

**Table 1.** Examples of Gene Neighborhoods Identified

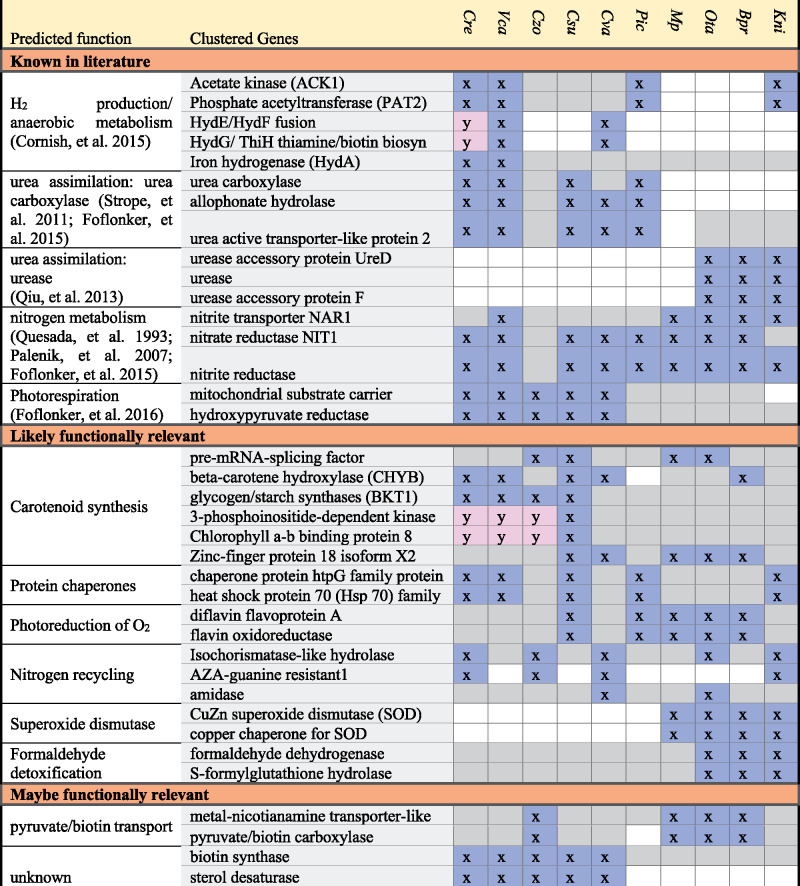

Note.—x and y are separate clusters. Gray indicates present but not in neighborhood. White indicates absence.

### Coexpression Analysis

To determine whether members in our captured neighborhoods are coexpressed, we utilized *C. reinhardtii* coexpression data from ALCOdb (Aoki et al. 2016). A total of 145 out of 317 gene neighborhoods comprised genes in the *C. reinhardtii* genome. A coexpression score was calculated as the sum of the pairwise mutual rank of the pairwise combinations of genes of a certain neighborhood size. A bootstrap analysis was performed with 5,000 simulated random neighborhoods of a certain size. Neighborhoods within the lowest 1% of coexpression scores of the simulated data were taken as significant (supplementary file 2, Supplementary Material online). Of those, 11 neighborhoods had significant coexpression scores (supplementary file 1, highlighted orange, Supplementary Material online), including the four neighborhoods that have been previously described in the literature.

### Green Algal Genomes Contain One of Two Putative Arsenic Detoxication Gene Clusters

Based on our neighborhood analysis, we identified two putative arsenic-detoxification pathways in green algae (fig. 2). One pathway present in *Chromochloris zofingiensis* (Chlorophyceaea), *Coccomyxa subellipsoidea* (Trebouxiophycea), and *K. nitens* (Streptophyta) may employ a strategy as described for bacteria. The three gene cluster contains homologs to the bacterial *arsBHC* operon. As described for nonalgal homologs, detoxification involves reduction of arsenate to arsenite by the arsenate reductase (ArsC), which can be exported by ArsB, known as ACR3 in fungi. Arsenite may also be methylated by ArsM (homologs found in all 9 algae, but not present in the arsenic gene neighborhoods, fig. 2), then oxidized by ArsH to a less toxic pentavalent form of methylated arsenate (Chen et al. 2015, 2017). Closely related homologs of the algal ArsB proteins are found in early diverging streptophytes and fungi, while the majority of bacterial homologs are from the Terrabacteria group, mainly Actinobacteria. Some closely related archaeal homologs were also identified (supplementaryfig. 4, Supplementary Material online). In contrast, homologs of algal ArsH are not found in land plants but are found in fungi. The most similar bacterial homologs are in Cyanobacteria and Proteobacteria (supplementary fig. 5, Supplementary Material online). Unlike the algal ArsB and ArsH families, the algal ArsC homologs are predicted to be more closely related to various bacterial homologs than to each other (supplementary fig. 6, Supplementary Material online). We also observed that the *C. zofingiensis* and *K. nitens ARS* genes have been recently duplicated. In *C. zofingiensis*, the entire 3-gene *ARS* cluster was duplicated, and gene order at both loci is maintained, while bordering genes are different (fig. 2*A*). In *K. nitens*, of the 6 *ARS* genes (2 paralogs for each gene), only a single 2-gene cluster is found.

**Fig. 4.**
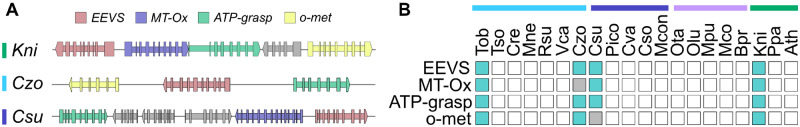
Putative MAA biosynthetic gene neighborhood. (*A*) Schematic of identified gene clusters. Gray gene models represent genes that do not have conserved proximity to the MAA gene cluster. (*B*) Phylogenetic profile of cluster members and homologs. Blue, present and clustered; gray, present but not clustered; white, absent. For all panels, the colored bars on the left designate taxonomic relationships, as indicated in figure 2.

**Fig. 5.**
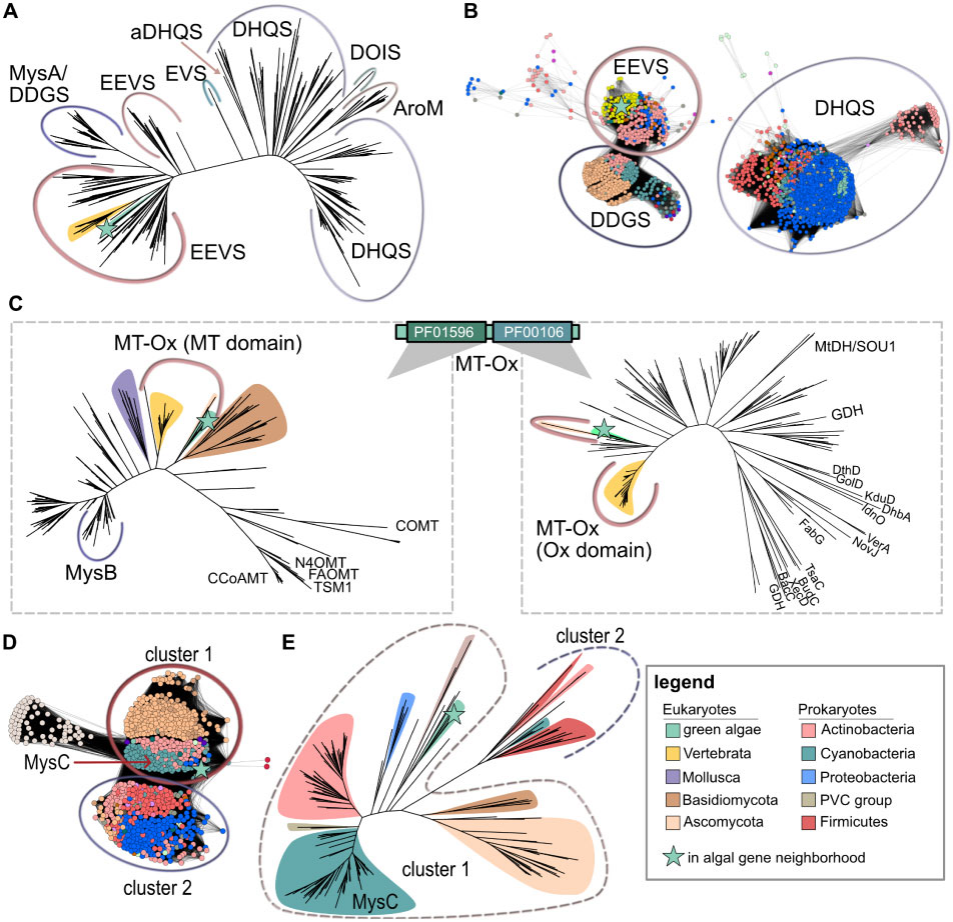
Phylogenetic and sequence similarity relationships of putative green algal MAA proteins. (*A*) Phylogenetic reconstruction of the sugar phosphate cyclase superfamily. The clade containing green algal EEVS orthologs are highlighted with a green background and green star. The closely related vertebrate clade is highlighted with yellow. (*B*) Sequence similarity network of proteins homologous to the putative algal EEVS proteins. DDGS- and EEVS-like proteins form two distinct clusters that are disconnected from the DHQS cluster. (*C*) Phylogenetic reconstruction of the methyltransferase (MT) domain (left) and the oxidase (Ox) domain (right). A cartoon of the protein’s domain organization is given with the respective Pfam domain IDs. Closely related eukaryotic clades are colored according to the legend. Enzyme names from SwissProt are shown as leaf labels. (*D*) Sequence similarity network of proteins homologous to the putative algal ATP-grasp-like protein. The circled clusters correspond to the outlined clades in panel *E*. (*E*) Phylogenetic reconstruction of proteins homologous to the algal ATP-grasp proteins. Clades corresponding to the clusters in panel *D* are outlined with a dotted line.

**Fig. 6.**
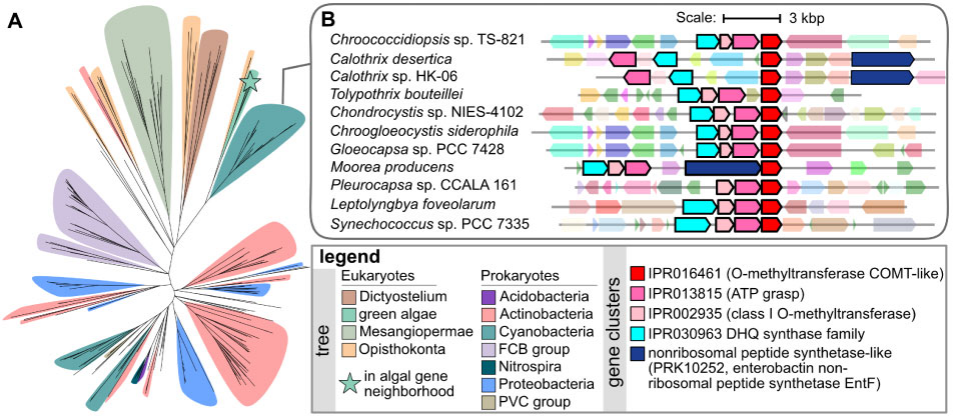
Phylogenetic and gene neighborhood analysis of the putative *o*-methyltransferase. (*A*) Phylogenetic reconstruction of proteins homologous to algal *o*-methyltransferase. Clades are colored by common taxonomy according to the legend. A star indicates the position of algal *o*-methyltransferase homologs identified in the MAA neighborhood. (*B*) Gene neighborhoods from the closely related cyanobacterial clade. Genes encoding likely MAA biosynthesis enzymes are represented with solid arrows with a black outline and colored according to legend.

In algal genomes that do not contain the *arsBHC*-type neighborhood, we identified a conserved neighborhood containing two genes annotated as glycolysis proteins, glyceraldehyde 3-phosphate dehydrogenase (GAPDH) and PGK, and a putative transporter belonging to the Major Facilitator Superfamily (MFS) (fig. 2*B*). Based on these annotations, one hypothesis is that this is a glycolysis-related cluster and the transporter may be involved in transporting products related to central carbon metabolism. However, a systematic analysis of homologous proteins suggests that the identified MFS-type transporter is related to ArsJ, a bacterial organoarsenical efflux permease, and bacterial homologs of the identified GAPDH that we refer to as GAPDH3, because it forms a distinct subfamily from either the plant-like GAPC/Cp or GAPA/B subfamilies, catalyze the formation of 1-arseno-3-phosphyglycerate from arsenate and glyceraldehyde 3-phosphate, which is then extruded from the cell via ArsJ (Chen et al. 2016) (fig. 3*A* and *B*). Not only are orthologs of these two proteins encoded by proximal genes in green algae, but they also cooccur in analyzed genomes; these genes are either both present or both absent (fig. 2*C*). The second putative glycolytic enzyme, PGK, is found proximal to the GAPDH3 and ArsJ genes in most but not all analyzed genomes (fig. 2*B* and *C*). Phylogenetic reconstruction of the PGK family suggests that these PGK homologs are distinct from canonical PGK enzymes (fig. 3*C*).

Green algae have four separate subfamilies of PGK-like proteins in addition to the canonical PGK proteins (fig. 3*C*). The paralog PGK2 is only found in Trebouxiophyceae and is distinct, but monophyletic, with canonical PGK. Although PGK2 genes only occur in algal genomes without a PGK3 or PGK4 ortholog, they are not proximal to GAPDH3 or ArsJ. The PGK3 homolog occurs in Chlorophyceae genomes, and the corresponding genes are found next to GAPDH3 and ArsJ genes with the exception of an ortholog in the trebouxiophyte *C. subellipsoidea*. The PGK3 family is similar to homologs from Actinobacteria, Chlamydia, and Deinococci. The PGK4 family is further split into two orthologous groups, PGK4A and PGK4B (fig. 3*C*). In green algae from Mamiellophyceae, PGK4A genes are, like PGK3, found next to either *ArsJ*, *GAPDH3*, or both, with the exception of homologs from early diverging streptophytes, such as *K. nitens* and *Selaginella moellendorffii*. In addition to PGK4, early diverging streptophytes have also a PGK4B that is composed of a fusion between the PGK-like domain PF00162 and a TrmB-like methyltransferase domain (PF02390). This fusion protein is the only PGK-like protein found in land plants (except *Arabidopsis thaliana*) outside of the canonical PGK subfamily.

### Identification of Gene Neighborhoods Leads to the Role of a PGK-like Protein in Resistance to Arsenic

Because of the complex phylogeny of noncanonical PGK-like proteins from algae and the presence of homologs in genomes that do not have the ArsJ-GAPDH detoxification route, the role of these PGK-like proteins in arsenate detoxification was ambiguous. Therefore, to test whether this gene functions in arsenate tolerance, we tested the sensitivity of a *C. reinhardtii PGK3* mutant to the presence of arsenate. A search of the publicly available sequenced mutant library of *C. reinhardtii* (Li et al. 2019) identified a strain with a marker inserted into *Cre07.g354250*, encoding PGK3, and a strain with a marker inserted into *Cre07.g354150*, encoding ArsJ (fig. 3*D*). Growth of the *pgk3* and *arsJ* mutants on agar-solidified medium was severely impaired in the presence sodium arsenate compared to the parent strain (fig. 3*E*).

### A Putative MAA Cluster

Several MAAs and related compounds have been found in cyanobacteria, algae, fungi, and vertebrates. These metabolites function as UV-absorbing “sunscreen” but may play other roles such as antioxidants or osmolytes (Oren and Gunde-Cimerman 2007). Four MAA-related genes were found to be cooccurring and clustered in three species, *K. nitens*, *C. zofingiensis*, and *C. subellipsoidea* (fig. 4). These species may have a hybrid biosynthetic pathway that contains enzymes related to either the vertebrate, as characterized in zebrafish to synthesize gadusol (Osborn et al. 2015), or cyanobacterial-like MAA pathways, as characterized for *Anabaena variabilis*, which has a 4-deoxygadusol intermediate (Balskus and Walsh 2010). The first step in both pathways is catalyzed by different sedoheptulose 7-phosphate cyclases, 2-epi-5-epi-valiolone synthase (EEVS) in vertebrates and desmethyl-4-deoxygadusol synthase (DDGS/MysA) in cyanobacteria (Gao and Garcia-Pichel 2011; Osborn et al. 2015). The algal neighborhood encodes a protein closely related to the vertebrate EEVS (fig. 5*A*). In addition to the phylogenetic reconstruction, the sequence similarity network of homologs clearly shows that the algal protein clusters with EEVS-like proteins and is distinct from DDGS/MysA-like proteins (fig. 5*B*). The second step of the vertebrate pathway is catalyzed by a protein that contains a methyltransferase and oxidase domains (Mt-Ox), whereas the cyanobacterial pathway involves a methyltransferase (MysB). The algal gene neighborhood encodes a double-domain protein closely related to the vertebrate Mt-Ox (fig. 5*C*).

Unlike the characterized vertebrate pathway, the algal gene neighborhood encodes two additional putative MAA synthesis enzymes, an ATP-grasp-like protein and an *o*-methyltransferase. The ATP-grasp protein is related to MysC from cyanobacteria (fig. 5*D* and *E*), which catalyzes the addition of an amino acid (such as glycine or serine) to 4-deoxygadusol. The putative *o*-methyltransferase is monophyletic with a clade of uncharacterized cyanobacterial proteins (fig. 6*A*). Although these proteins are uncharacterized, corresponding cyanobacterial genes are often found proximal to genes encoding homologs of MysA, MysB, MysC, and a nonribosomal peptide synthetase (fig. 6*B*), suggesting that the cyanobacterial protein and, by extension, the algal proteins are also involved in the synthesis of a MAA. Together, these analyses lead to the hypothesis that these green algae may synthesize a MAA that has a gadusol core instead of the deoxygadusol core found in cyanobacterial MAAs.

### Evolutionary Trends

The closest nongreen algal homologs were identified for 755 genes in the 317 neighborhoods by manual and automated sorting (PhysortR [Stephens et al. 2016]) of phylogenetic trees (IQ-TREE [Minh et al. 2020]) built from the top 50 BLASTp hits. The majority, 63%, of proteins are plant-like, meaning that the closest nonchlorophyte homologs are in the land plant lineage. Twelve percent of proteins have detectable homologs in only other green algal genomes or had too few detectable homologs to build a tree. Prokaryotic (5%) and metazoan (3%) homologs (i.e., proteins that appeared to be more closely related to prokaryotes or metazoans than other lineages) represent smaller percentages (supplementary fig. 7*A*, Supplementary Material online). At the neighborhood level, 40% of neighborhoods encode only streptophyte-like proteins, 14% encode proteins with Viridiplantae homologs (streptophyte-like proteins and proteins only detected in other chlorophytes), and 2% contained only chlorophyte-specific proteins. Mixed clusters comprised proteins that did not have homologs in the same lineages (outside Chlorophyta) were 16% of total clusters (supplementary fig. 7*B*, Supplementary Material online). Only one neighborhood encoded proteins that had non-Viridiplantae homologs: two cyanobacterial-like proteins, an iron-containing redox family protein and a putative SAM-dependent methyltransferase. The streptophyte- and Viridiplantae-like neighborhoods suggest that most genes in the identified neighborhoods were in the last common green ancestor and maintained during green algal and land plant evolution. If HGT of whole neighborhoods occurred, then those events are ancient (i.e., would have to of occurred in a green algal ancestor of the chlorophytes and land plants).

## Discussion

Using a comparative genomic approach that is independent of a priori knowledge of gene function, we have identified conserved gene clusters in green algal genomes. A total of 317 gene neighborhoods were identified, 64–92 gene neighborhoods per species, representing between 1.2% and 2.8% of chlorophyte genes, with 0.5–1.9% of genes in neighborhoods conserved in the streptophyte alga *K. nitens*. Since our method relies on conservation of gene proximity in evolutionarily distant genomes, the availability of more high-quality algal genomes will likely result in the identification of more gene clusters. Indeed, a recent analysis of 341 fungal genomes found 1,704 cluster families present in at least two different taxonomic classes (allowing for three intervening nonhomologous genes) (Marcet-Houben and Gabaldón 2020). Therefore, the green algal neighborhoods identified in this analysis using ten algal genomes should not be considered the extent of gene clustering and conservation in green algae.

We did find that gene neighborhoods are widely distributed and conserved among the green algal species examined here. Our analysis captured known green algal neighborhoods involved in nitrate metabolism, photorespiration, and urea metabolism, providing support for the ability of this strategy to identify cofunctional gene clusters (Quesada et al. 1993; Strope et al. 2011; Qiu et al. 2013; Foflonker et al. 2016). The majority of neighborhoods identified, however, contained unannotated genes. Of particular interest are neighborhoods containing a mixture of well annotated and poorly or unannotated genes, which could serve to inform on the function of unannotated genes. However, as demonstrated by the prevalence of semantically distant Gene Ontology terms for colocated genes in prokaryotic, fungal and metazoan gene clusters (Mihelčić et al. 2019), how genes are functionally linked is not always clear, and accurate predictions are not easily automated. Therefore, we implemented a detailed phylogenomic approach that led to the discovery of two arsenic detoxification neighborhoods and a putative MAA biosynthetic cluster.

Arsenic is a prevalent toxin in the environment, and multiple mechanisms for arsenic tolerance have been described for algae. These mechanisms include adsorption on the cell surface, vacuole sequestration, complexation with thiols, methylation, excretion, reduction, and transformation into organoarsenic compounds (Wang et al. 2015). Our analysis identified two gene neighborhoods encoding members with similarity to known arsenic detoxification enzymes. The first is similar to the *arsBHC* operon found in *Synechocystis* containing an arsenate reductase, transporter, and a gene involved in the arsenic methylation pathway (Wang et al. 2015). A second pathway found in species lacking the first neighborhood contains a bacterial-like GAPDH homolog and a transporter homologous to ArsJ, which are encoded in bacterial *ars* operons. Together, these proteins function as an organoarsenical efflux system in *Pseudomonas aeruginosa* (Chen et al. 2016). While these genes are not found in available land plant genomes, an analogous pathway involving GAPDH and a transporter, with similar function to ArsJ but arose by convergent evolution, was recently described for the arsenic-hyperaccumulating fern *Pteris vittata* (Cai et al. 2019). Algal GAPDH3 and ArsJ were likely acquired through HGT from a bacterium, while the GAPDH and organoarsenic transporter in *P. vittata* involved in arsenate detoxification are members of the plant GAPC and OCT4 families, respectively. Therefore, if the algal pathway was found in the green algal ancestor of land plants, it was lost, and an analogous pathway re-evolved in *P. vittata.*

In algae, homologs of the bacterial *ars* GAPDH and ArsJ are accompanied by a second glycolytic gene encoding PGK that was not formerly known to functionally cooperate with the organoarsenical efflux system, suggesting that the PGK neighbor is an alga-specific adaptation. Phylogenetic analysis of the PGK family suggests that the neighboring PGKs form two subfamilies distinct from the canonical PGKs, with PGK3 conserved among core chlorophytes, and PGK4 conserved among prasinophytes and streptophytes. The PGK4 subfamily is further divided into a clade that contains prasinophytes and early diverging streptophytes and a clade that contains streptophytes (not *A. thaliana*). The later clade contains proteins, represented by LOC_Os10g30550 in rice, that are composed of an N-terminal domain homologous to PGK and a C-terminal domain homologous to a TrmB-like methyltransferase. The function of PGK4 and the fusion protein are unknown, but the link to arsenic detoxification based on our neighborhood analysis and the sensitivity of a *C. reinhardtii* PGK3 mutant to arsenate opens the possibility, that like PGK3, PGK4 homologs may be involved in arsenic detoxification.

Although we observed that the mutant carrying an insert in the *PGK3* gene displayed increased arsenate sensitivity, the enzymatic role of PGK3 is not known. One hypothesis is that like bacterial *ars* GAPDH (Chen et al. 2016), algal GAPDH3 catalyzes arsenylation of G3P to form 1As3PGA that is unstable and may spontaneously hydrolyze into As(V) and 3PGA before 1As3PGA can be transported out of the cell by algal ArsJ. Under this scenario, PGK3 could function in concert with GAPDH3 to recycle the resulting 3PGA producing G3P. The cost of an ATP and NADPH to perform the recycle and avoid the build-up of 3PGA may be advantageous for these photosynthetic microbes, where the artificial build-up of 3PGA, due to spontaneous hydrolysis of 1As3PGA, may inadvertently stimulate starch synthesis (Ball et al. 1991).

In addition to the arsenic-detoxification pathways, we also found a putative MAA biosynthetic cluster among the identified gene neighborhoods. The green algal MAA synthesis genes are found clustered in at least four green algal genomes (fig. 4) and may synthesize a MAA with a gadusol core instead of the deoxygadusol core that is common for cyanobacterial MAAs. The first two proteins in the green algal pathway are related to vertebrate EEVS and Mt-Ox, which function together to make gadusol. The remaining two proteins encoded by the neighborhood may be involved in the attachment of an amino acid to gadusol (catalyzed by a homolog of MysC) and subsequent methylation (catalyzed by a putative *o*-methyltransferase that is related to an *o*-methyltransferase often encoded by uncharacterized cyanobacterial MAA gene clusters). Although the functions of these algal enzymes have yet to be experimentally tested, a MAA with a gadusol core from the green alga *Prasiola calophylla*, which has an absorption maximum at 324 nm, named prasiolin has been previously isolated (Hartmannet al. 2016). A distinct MAA, also with an absorption maximum at 324 nm, is found in various *Klebsormidium* species (Kitzing and Karsten 2015). Although the chemical structures are distinct from prasiolin, these MAAs, klebsormidin A and klebsormidin B, also contain a gadusol core (Hartmann et al. 2020). Since klebsormidin A, but not klebsormidin B, was isolated from a strain of *K. nitens*, the identified gene neighborhood in *K. nitens*, *C. zofingiensis*, and *C. subellipsoidea* may be responsible for klebsormidin A synthesis. In addition, synthetic MAAs, named gadusporines, have been created by recombinant expression of vertebrate gadusol biosynthetic genes with bacterial *mysC* and *mysD* genes (Osborn and Mahmud 2019). Therefore, this conserved green algal neighborhood likely represents a naturally evolved hybrid between the vertebrate and bacterial pathways that produces a MAA with a gadusol core, but further experimentation is needed to confirm the identity of the MAA compound produced by this pathway.

The specific selective advantage for maintaining these gene neighborhoods is not readily known and likely varies between neighborhoods from epistatic selection to epigenetic regulation. Gene neighborhoods in eukaryotes may have formed through genomic rearrangement, neofunctionalization, or HGT of whole pathways (Wisecaver and Rokas 2015; Nützmann et al. 2018). Coinheritance of advantageous genes, an effort to avoid toxic intermediates (Wong and Wolfe 2005; McGary et al. 2013), or selection for coexpressed genes may drive the maintenance of this type of genomic organization (Nützmann et al. 2016). Given that the last common ancestor of the chlorophyte and streptophyte lineages existed at least 800–1,000 Ma (Blaby-Haas and Merchant 2019) many of these neighborhoods, like the putative MAA pathway, are ancient (assuming that HGT has not occurred between these green algal genomes). Conservation of gene proximity suggests selective advantage. The majority of genes in neighborhoods have closely related homologs in land plants, indicating vertical inheritance, possibly followed by genome rearrangement or duplication and neofunctionalization of genes.

Little evidence that the gene neighborhoods we identified evolved by HGT of intact gene clusters from nongreen algae was found (as described for the “selfish operon model” [Lawrence and Roth 1996]). However, this does not preclude the possibility of HGT of individual genes recruited into neighborhoods or ancient transfer events that are not detectable. For instance, we hypothesize that GAPDH and ArsJ were likely acquired as a functional unit from bacteria, but PGK3 was subsequently recruited. As an example of the toxic intermediate hypothesis, intermediates of ArsBHC are more toxic than arsenate suggesting that toxin tolerance could act as a selective pressure that favors gene clustering, and arsenic resistance genes are also clustered in yeast (Bobrowicz et al. 1997). Additional evidence for de novo assembly of gene neighborhoods in eukaryotes has been seen in triterpene pathways in plants, the mycotoxin trichothecene in fungi, and the DAL cluster, involved in the conversion of allantoin to urea in yeast (Wong and Wolfe 2005; Field and Osbourn 2008; Proctor et al. 2009). Gene clustering analysis in fungi points to vertical evolution and differential loss as the dominant evolutionary mechanism for clustering followed by convergent evolution (Marcet-Houben and Gabaldón 2019). Other examples of convergent evolution include the GAL cluster in fungi, which is predicted to have originated through de novo assembly and HGT in different species (Rokas et al. 2018). From a comparative genomics perspective, analysis of conserved gene clustering may provide insights into shared selective pressures between these species. Questions remain as to what mechanism is providing a selective advantage resulting in some neighborhoods to be maintained in certain lineages and not others.

## Materials and Methods

### Identifying Conserved Gene Neighborhoods

A perl script (available from: https://github.com/ffoflonker/gene-neighborhoods) was used to identify clusters of conserved orthologous genes in close proximity among nine chlorophyte green algae *Volvox carteri* v2.1 (Prochnik et al. 2010), *C. reinhardtii* v5.5 (Blaby et al. 2014), *C. zofingiensis* (Roth et al. 2017) (updated annotation available from: https://sites.google.com/view/czofingiensis/home), *C. subellipsoidea* C-169 v2.0 (Blanc et al. 2012), *Picochlorum* SENEW3 v2.0 (Foflonker et al. 2018), *Chlorella* sp. NC64 (Blanc et al. 2010), *Ostreococcus tauri* RCC4221 v3.0 (Palenik et al. 2007), *Micromonas pusilla* CCMP1545 v3.0 (Worden et al. 2009), *Bathycoccus prasinos* v1.0 (Moreau et al. 2012). OrthoFinder (Emms and Kelly 2015) was used to identify orthologous groups among the 9 chlorophytes and *K. nitens* v1.1 (formerly *Klebsormidium flaccidum*) (Hori et al. 2014). Gene neighborhoods were identified based on a set minimum number of species with POG pairs within a set window size (gene number). A minimum of two POG pairs was required to denote a neighborhood. Window size was used to approximate a sequence length in which to search for POG pairs and is equal to the average gene size per chromosome multiplied by the window size. A window size of 6 was chosen for this analysis. Overlapping windows were merged. Larger window sizes may result in multiple neighborhoods in one window, which was not separated in this analysis. Gene neighborhoods were then ranked, giving weight to neighborhoods containing genes with smaller orthologous groups. This was done by dividing the number of clustered genes in an orthologous group by the number of total genes in the orthologous group. This was then divided by the number of genes in the neighborhood and multiplied by 10. This ranked list was then filtered for unique neighborhoods by removing any neighborhoods containing any genes already present in a neighborhood with higher ranking. A final clean-up step was performed to remove any genes that did not meet the search criteria, highlighting only the POG pairs. Gene clustering statistics were reported from this list and excluded histone gene neighborhoods.

Gene neighborhood size is defined as the number of POG pairs greater than or equal to the set minimum ortholog number (criterion 1). Additional gene neighborhoods were identified by searching for neighborhoods containing only cooccurring proximal genes (criterion 3) (i.e., POG pairs are clustered in every genome in which they are present), or proximal genes with more distant conserved orthology to the streptophyte alga, *K. nitens* (criterion 2). These two searches were performed with relaxed the search parameters (min. orthologs = 3).

### Annotation

Blast2GO (Conesa et al. 2005) was used to automatically annotate neighborhoods, then neighborhoods were manually annotated and inspected for potential functional relevance. Transporter classification database (Saier Jr et al. 2006) was used to annotate transporters. The Enzyme Function Initiative Enzyme Similarity Tool (EFI-EST) and Enzyme Function Initiative Genome Neighborhood tool (EFI-GNT) were used to identify similar gene neighborhoods in bacteria (Gerlt et al. 2015). Identified neighborhoods were also used to search for conservation of proximal genes in a broader list of algal species. Gene IDs of mentioned gene neighborhoods available in supplementary table 1, Supplementary Material online.

### Coexpression Analysis

Coexpression data for the *C. reinhardtii* genome was downloaded from ALCOdb (Aoki et al. 2016). The sum of the pairwise mutual rank for each pairwise combination of genes (removing genes from the same orthologous group) in a neighborhood of a certain size was used as the coexpression score. Pairwise mutual ranks >1,000 were estimated as 1,000. A bootstrap analysis pulling 5,000 random gene neighborhoods of a particular size was done to generate neighborhood coexpression score distributions. Neighborhoods with scores within the lowest 1% of simulated data were taken as significant (neighborhood size 2, coexpression score cutoff: 168; size 3, cutoff: 2,052; size 5, cutoff: 8,191). Data available in supplementary file 2, Supplementary Material online.

### *Chlamydomonas reinhardtii* Mutant Screens

*C. reinhardtii* CliP mutants (LMJ.RY0402. 109886 and LMJ.RY0402.043400) and wild-type strain CC-5325 were ordered from the *Chlamydomonas* Resource Center. Cultures were maintained in Tris-Acetate-Phosphate (TAP) agar plates and liquid culture under continuous light. Agar (Invitrogen Select Agar) was washed to remove impurities. Growth screens were performed by suspending actively growing cells in liquid TAP to the same cell densities and spotting equal volumes of each suspension onto TAP agar plates without or with sodium arsenate (10–320 µM). Plates were incubated at 25 °C and 50 µE m^−2^ s^−1^.

### Sequence Similarity Networks and Family-Specific Phylogenetic Analyses

The EFI-EST was used to build similarity networks. For ArsJ network, BLASTp with ARSJ from *C. reinhardtii* as the query was used to retrieve 1,513 sequences; an alignment score of 90 was used for defining edges. For GAPDH network, the InterPro domain IPR020830 was used to retrieve representative UniRef90 cluster sequences; an alignment score of 100 was used for defining edges; nodes were collapsed at 60% similarity. For the EEVS network, BLASTp with Cz11g05100 (Cz_Braker2|chr11.g12594.t1) from *C. zofingiensis* as the query was used to retrieve 5,000 sequences; an alignment score of 70 was used for defining edges. For the ATP-grasp network, BLASTp with Cz11g05110 (Cz_Braker2|chr11.g12595.t1) from *C. zofingiensis* as the query was used to retrieve 1,507 sequences; an alignment score of 35 was used for defining edges.

For the phylogenetic analyses, homologous protein sequences were retrieved from SwissProt and combined with homologous proteins representing UniRef90 clusters. Multiple sequence alignments were built using MAFFT (Katoh and Standley 2013). Phylogenetic trees were built using FastTree (Price et al. 2010) on the CIPRES Science Gateway (Miller et al. 2010) and visualized with iTOL (Letunic and Bork 2019); branches with a bootstrap value less than 0.5 (based on 1,000 bootstrap replicates) were deleted. Protein IDs, multiple sequence alignments, and trees in Newick format can be found in supplementary files 3 and 4, Supplementary Material online.

### Identifying Evolutionary Trends

A BLASTp search was performed for 755 genes (one representative gene per orthologous group) of the 317 clusters identified against NCBI’s RefSeq nonredundant protein database. Sequences from the top 50 hits (smallest *E* values) were retrieved and aligned using Muscle (Edgar 2004). IQ-TREEs were then generated for each using default parameters and an ultrafast bootstrap value of 1,000 (Minh et al. 2020). PhysortR was used to categorize trees based on closest nongreen algal relatives, nonexclusive clades were manually examined (Stephens et al. 2016). Manual curation was used on difficult to determine trees. The green algal-specific classification was given to trees with only green algae and genes with too few Blast hits to create a tree. Gene neighborhoods were then classified based on the closest homolog determination of the genes included in the neighborhood. The “unknown” category was given to neighborhoods with one gene categorized as having an unknown closest homolog, unless the neighborhood already contained genes with different closest homologs, it was then categorized as “mixed.”

## Supplementary Material

Supplementary data are available at *Molecular Biology and Evolution* online.

## Supplementary Material

msaa221_Supplementary_Data
