# Supplementary material for: Colocality to Cofunctionality: Eukaryotic Gene Neighborhoods as a Resource for Function Discovery
Source: Mol Biol Evol. 2020 Sep 4;38(2):650–62. doi: 10.1093/molbev/msaa221 (PMC7826186; doi:10.1093/molbev/msaa221)
Supplement: msaa221_Supplementary_Data [file msaa221_supplementary_data.zip › msaa221-suppl_data/SI_Figures.pdf]

Supplementary Information for Co-locality to co-functionality: Eukaryotic gene neighborhoods as a resource for function discovery

SI Figure 1. Method overview

SI Figure 2. Number and percentage of genes in gene neighborhoods based on individual criteria

SI Figure 3. Number of gene neighborhoods identified using different window sizes with a minimum ortholog number set to 4

SI Figure 4. ArsB/ACR3-like protein family

SI Figure 5. ArsH-like protein family

SI Figure 6. ArsC-like protein family analysis

SI Figure 7. Analysis of closest homologs

SI Table 1. Gene IDs of mentioned gene neighborhoods

SI File 1 (separate excel file). List of all gene neighborhoods and putative annotations

SI File 2 (separate excel file). Coexpression analysis. Coexpression scores of the 145 neighborhoods containing *C. reinhardtii* genes and the random sampling analyses

SI File 3 (separate excel file). Arsenic detoxification neighborhood protein information

SI File 4 (separate excel file). MAA biosynthetic cluster protein information

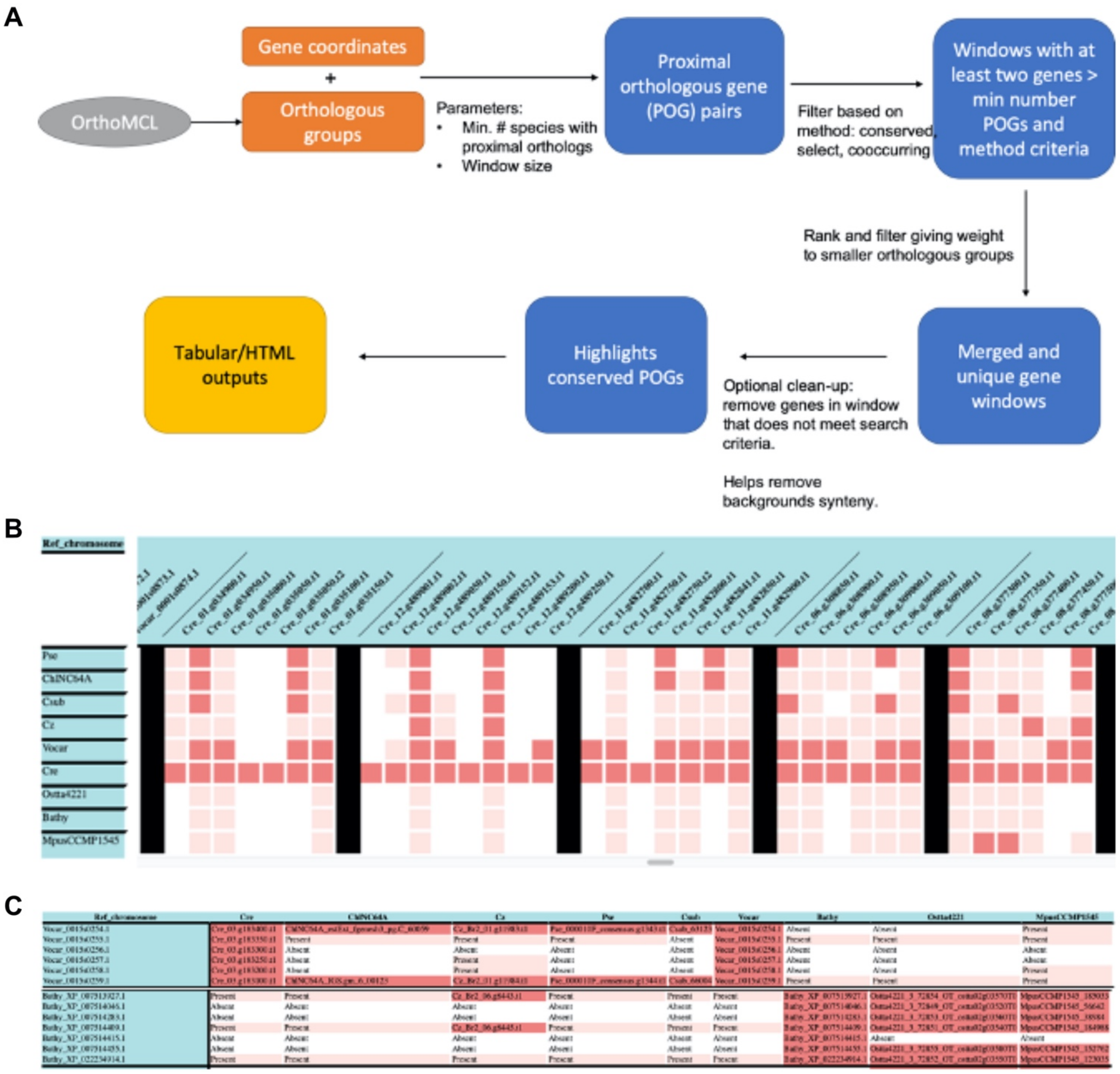

SI Figure 1. Method overview (A) and sample outputs (B, C). In the outputs, red are genes that are proximal and conserved between genomes, light red indicates genes that have homologs which are not proximal in both genomes, and white represents an absence of a homologous gene in the second genome. Output B can be used to visualize evolutionarily conserved genes with the reference backbone along the top row and the ability to scroll across all gene neighborhoods identified. Output table C shows clusters with gene ID information and the reference backbone in the first column.

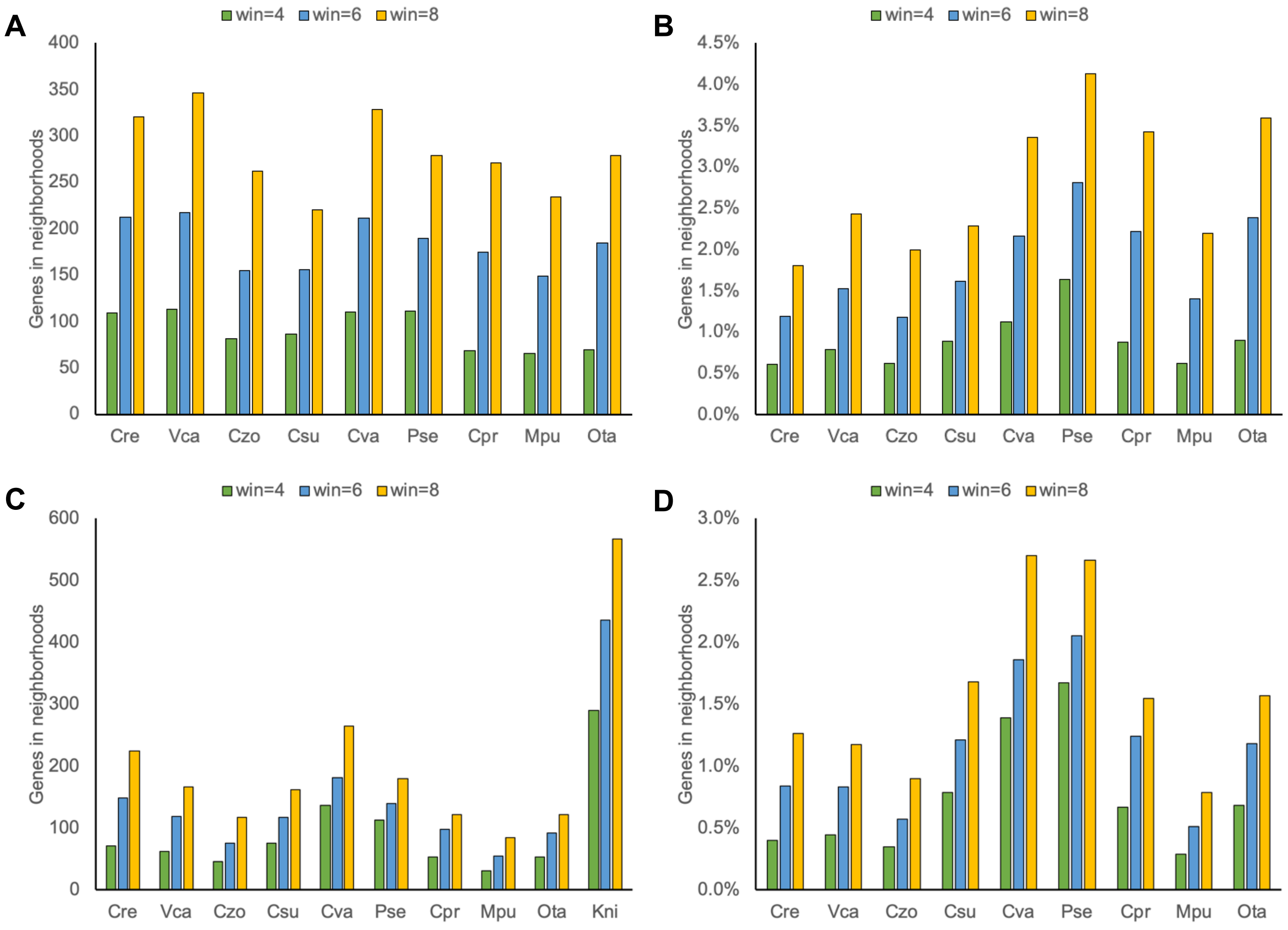

SI Figure 2. Number (A) and percentage (B) of genes in gene neighborhoods in various window sizes using criteria 1. Total gene neighborhoods: 85 (win=4), 152 (win=6), and 204 (win=8). Number (C) and percentage (D) of genes in gene neighborhoods in various window sizes conserved with *Klebsmoridium nitens* (Kni) (criteria 2). Total gene neighborhoods: 119 (win=4), 175 (win=6), and 202 (win=8). Vca: *Volvox carterii*, Cre: *Chlamydomonas reinhardtii*, Czo: *Chromochloris zofingiensis*, Csu: *Coccomyxa subellipsoidea*, Pse: *Picochlorum* SENEW3, Cva: *Chlorella* sp. NC64, Mpu: *Micromonas pusilla*, Ota: *Ostreococcus tauri*, Bpr: *Bathycococcus prasinos*.

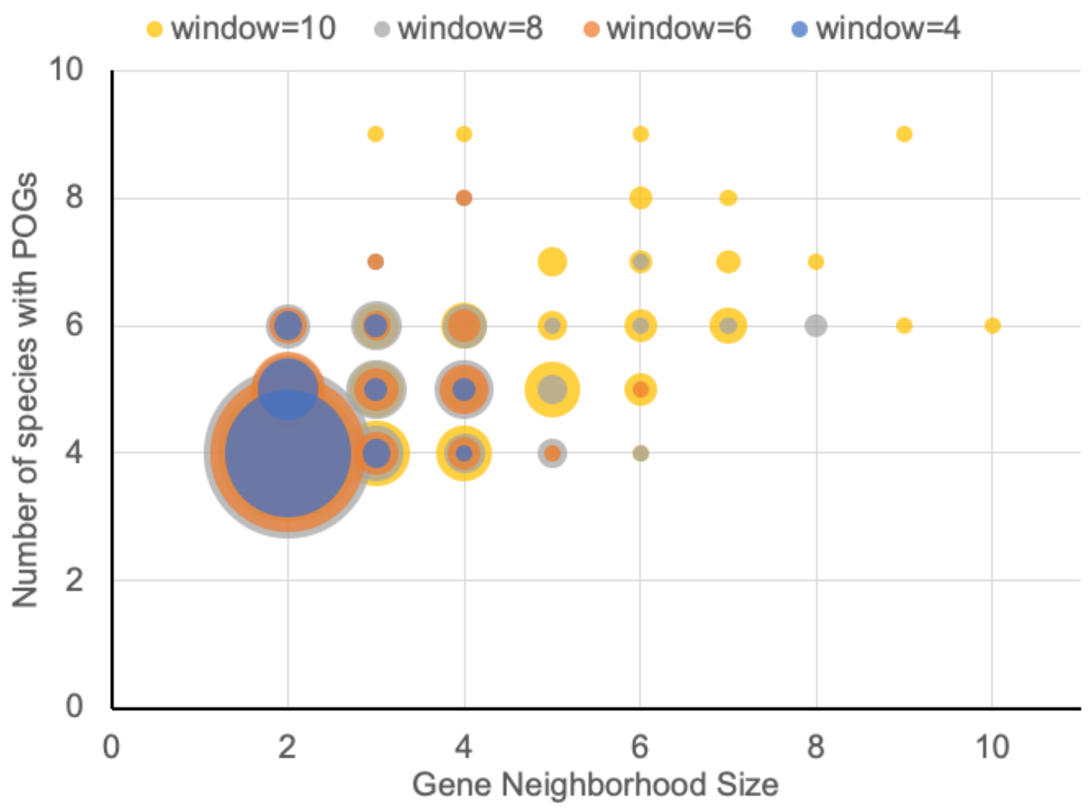

SI Figure 3. Number of gene neighborhoods identified using different window sizes with a minimum ortholog number set to 4. The total number of neighborhoods are 85, 152, 204, and 189 for window sizes 4, 6, 8, and 10 respectively. Gene neighborhood size is defined as the number of conserved proximal orthologous genes (POGs) with greater than the minimum ortholog number of species (criterium 1). Largest and smallest bubbles sizes represent 105 and 1 neighborhood. Larger window sizes (>9), result in merging of multiple smaller clusters as indicated by decrease in gene neighborhood number and increase in gene neighborhood size.

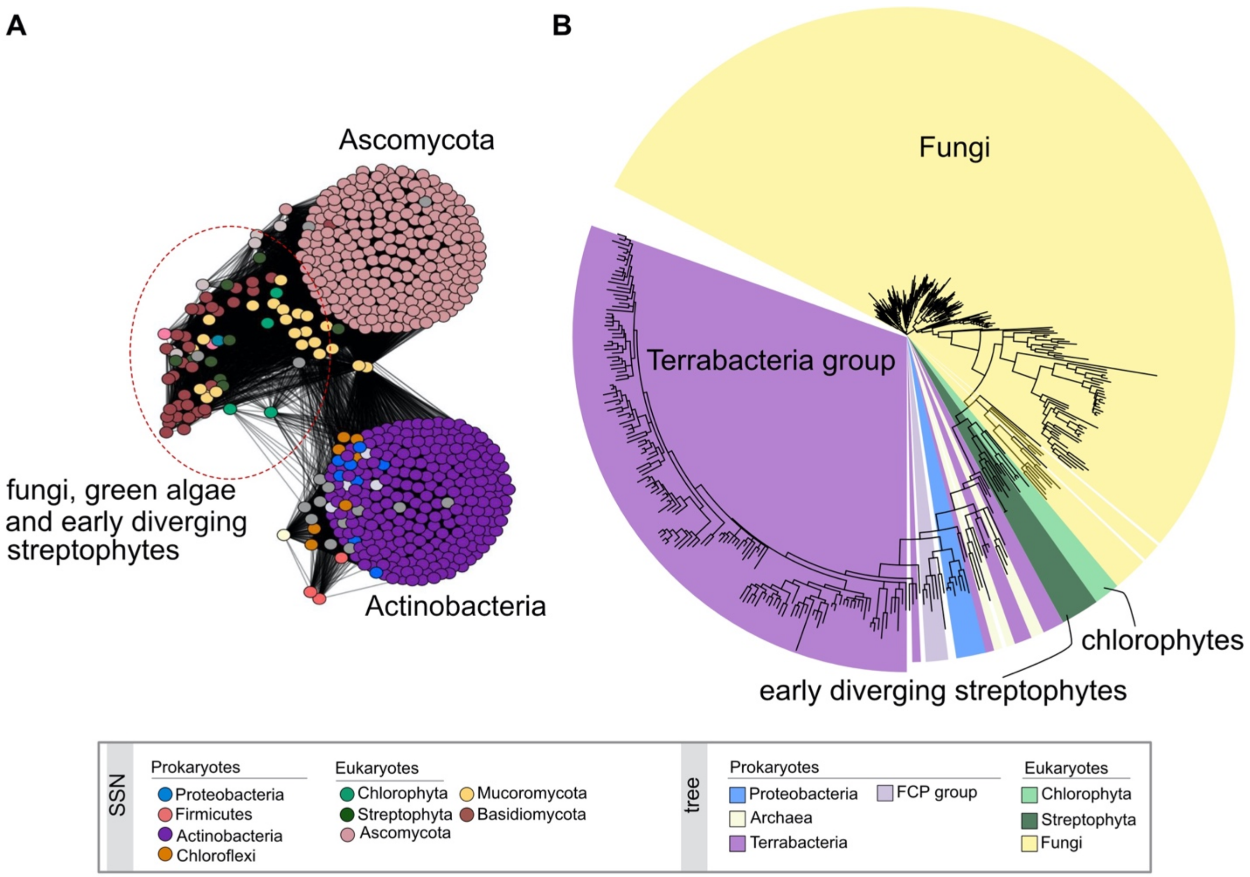

SI Figure 4. ArsB/ACR3-like protein family. (A) SSN of proteins similar to the algal ArsB-like proteins encoded by the arsenic-related neighborhood. Nodes are colored by phylum. (B) Phylogenetic reconstruction of proteins similar to the algal ArsB-like proteins encoded by the arsenic-related neighborhood. Clades are shaded by taxonomic grouping, as indicated.

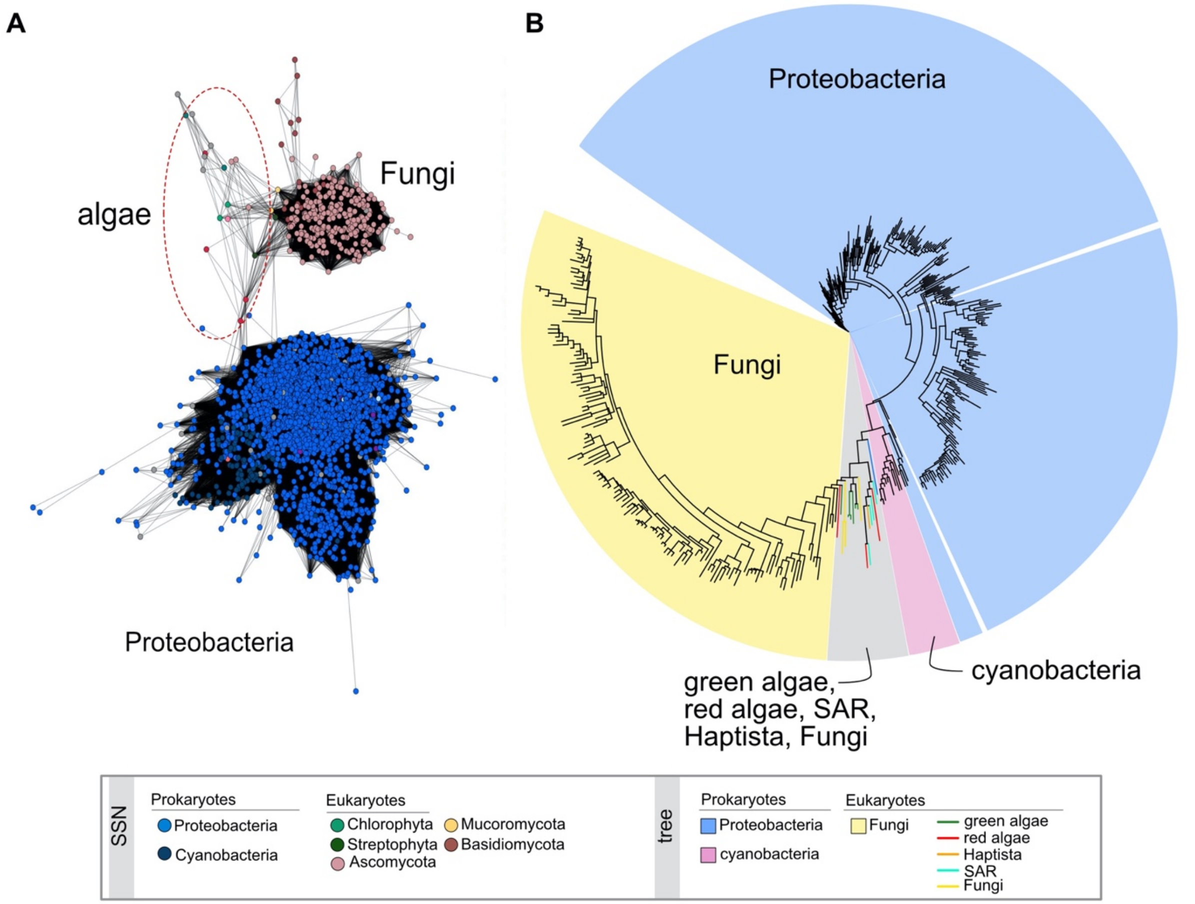

SI Figure 5. ArsH-like protein family. (A) SSN of proteins similar to the algal ArsH-like proteins encoded by the arsenic-related neighborhood. Nodes are colored by phylum. (B) Phylogenetic reconstruction of proteins similar to the algal ArsH-like proteins encoded by the arsenic-related neighborhood. Clades are shaded by taxonomic grouping, as indicated, with the exception of the clades containing algal sequences, which are shaded grey, because they contain multiple taxonomic groupings.

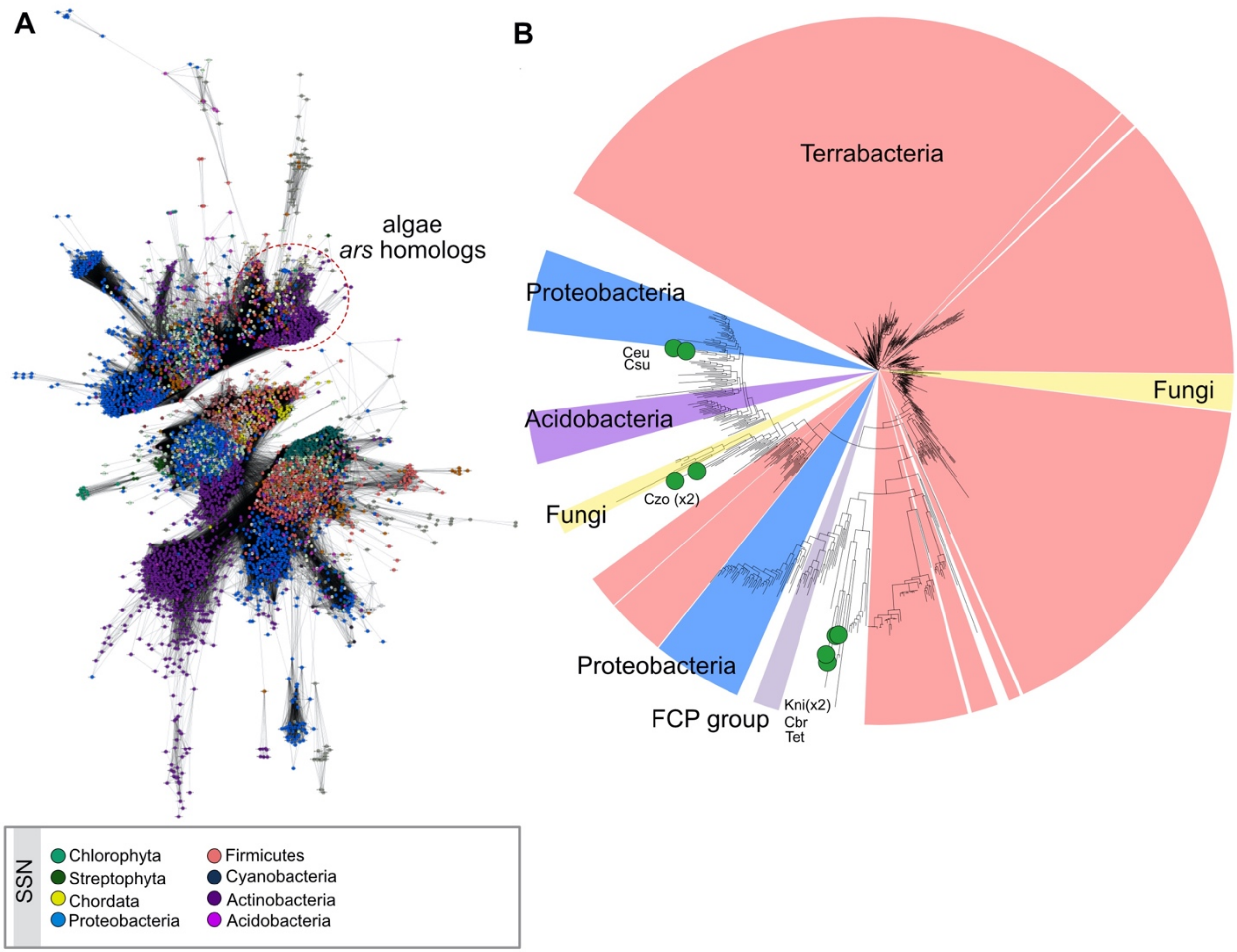

SI Figure 6. ArsC-like protein family analysis. (A) SSN was made with sequences containing the PF01451 domain. Each node represents a UniRef50 cluster. Alignment score of 20 was used. Nodes are colored by the phylum of the representative sequences for each UniRef50 cluster. (B) Phylogenetic reconstruction of proteins similar to the algal ArsC-like proteins encoded by the arsenic-related neighborhood. Clades are shaded by taxonomic grouping, as indicated. Leaves corresponding to algal sequences are indicated with a green circle. Except for most of the Terrabacteria-containing clades, the clades tend to not to have a common taxonomic level other than Bacteria, and are not shaded.

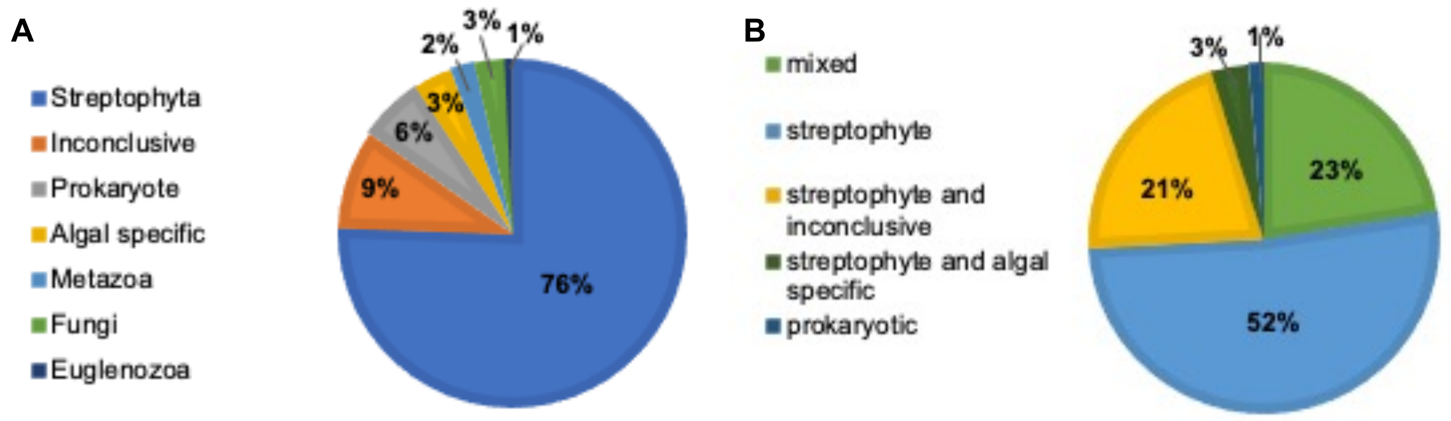

SI Figure 7. Homolog analysis. (A) Proportion of total proteins with closest homologs in the indicated lineage. (B) Proportion of neighborhoods containing neighbors whose closest homologs are from the same lineage or different lineages.

SI Table 1. Gene IDs of mentioned gene neighborhoods. Red genes are clustered; black represents non clustered genes.

	Cre	Vca	Czo	Csu	Cva	Pse	Mpu	Ota	Bpr	Kni
**ArsJ**	Cre07.g354150	Vocar.0017s0059	N/A	N/A	gw1.9.78	000004F_consensus.g936	23811	ostta02g02850	007514191	N/A
**PGK**	Cre07.g35425, Cre11.g467770	Vocar.0017s0058, Vocar_0030s0188	Cz_Braker2|chr16.g10819.t1	7028, 53354	estExt_Genewise1.C_20504,fgenesh3_pm.C_scaffold_2000012	000008F_consensus.g1443	157150	ostta02g02860, ostta06g00700	007514126	kfl00038_0330_v1
**GAPDH3**	Cre07.g354200	Vocar.0017s0057	N/A	N/A	IGS.gm_9_00189	000004F_consensus.g935	49771	ostta02g02850	007514097	N/A
**CrACR2**	Cre07.g354300 Cre01.g02485 Cre05.g24740 Cre07.g352550	Vocar.0005s0089, Vocar.0007s0372, Vocar.0082s0003	Cz_Braker2|chr04.g5147.t1	66432	IGS.gm_5_00094	N/A	N/A	N/A	N/A	kfl00445_0080_v1
**ArsH**	N/A	N/A	Cz_Braker2|chr09.g9571.t1 Cz_Braker2|chr17.g2488.t1	28192	N/A	N/A	N/A	N/A	N/A	kfl00111_0020_v1
**ArsB**	N/A	N/A	Cz_Braker2|chr09.g9572.t1 Cz_Braker2|chr17.g2489.t1	14170	N/A	N/A	N/A	N/A	N/A	kfl00534_0030_v1, Kfl00804_0040_v1
**ArsC**	N/A	N/A	Cz_Braker2|chr09.g9573.t1 Cz_Braker2|chr17.g2490.t1	Found in nuclear sequence scaffold_5:513481-514193	N/A	N/A	N/A	N/A	N/A	kfl00111_0010_v1
**EEVS**	N/A	N/A	Cz_Braker2|chr11.g12594.t1	39261	N/A	N/A	N/A	N/A	N/A	kfl00216_0060_v1
**MT-ox**	N/A	N/A	Cz_Braker2|chr03.g4211.t1	55647	N/A	N/A	N/A	N/A	N/A	kfl00216_0070_v1
**MysC**	N/A	N/A	Cz_Braker2|chr11.g12595.t1	55651	N/A	N/A	N/A	N/A	N/A	kfl00216_0080_v1
**o-met**	N/A	N/A	Cz_Braker2|chr11.g12595.t1	55651	N/A	N/A	N/A	N/A	N/A	kfl00216_0100_v1
